# Developments in Toxicity Testing with Duckweeds

**DOI:** 10.3390/jox15020048

**Published:** 2025-03-26

**Authors:** Paul Ziegler

**Affiliations:** Department of Plant Physiology, University of Bayreuth, 95440 Bayreuth, Germany; paul.ziegler@uni-bayreuth.de

**Keywords:** duckweeds, toxicity tests, biomarkers

## Abstract

Duckweeds are a family of small floating macrophytes (the Lemnaceae) that inhabit quiet freshwaters worldwide. They have long been employed to determine toxicity to higher plants in the aquatic environment, and standardized national and international protocols have been developed for this purpose using two representative species. While these protocols, which assess the growth of the leaf-like fronds of the tested duckweed, are indeed suitable and still frequently used for detecting the toxicity of water-borne substances to aquatic higher plant life, they are cumbersome and lengthy, determine endpoints rather than depict toxicity timelines, and provide no information as to the mechanisms involved in the indicated toxicity. Progress has been made in downscaling, shortening and improving the standardized assay procedures, and the use of alternative duckweed species, protocols and endpoints for detecting toxicity has been explored. Biomarkers of toxic effect have long been determined concomitantly with testing for toxicity itself, and their potential for the assessment of toxicity has recently been greatly expanded by transcriptomic, proteomic and metabolomic techniques complemented by FITR spectroscopy, transformation and genotoxicity and timescale toxicity testing. Improved modern biomarker analysis can help to both better understand the mechanisms underlying toxicity and facilitate the identification of unknown toxins.

## 1. Introduction

The influx of industrial, agricultural, municipal and domestic waste into natural waters poses serious hazards for life forms inhabiting or consuming these waters, including bacteria, plants, invertebrates, fish and mammals, including man [1,2]. The study of the effects of water pollutants on aquatic plant life is particularly important from the viewpoint of plants being primary producers and thus supportive of other aquatic organisms along the food chain and that they also pass on toxic substances that they may have taken up to organisms consuming them. Aquatic plant life includes micro- and macroalgae, bryophytes, pteridophytes and flowering plants, species of each of which are used in testing for deleterious effects of water pollutants [2]. Microalgae have long been the most frequently used life form for investigating the effects of water contaminants on aquatic plant life (see [2,3]). However, flowering plants come next in this regard [2], most prominently rooted species of the genus *Myriophyllum* (water milfoils) and duckweeds [2], a widespread family of small, simply constructed floating macrophytes (the Lemnaceae) that inhabit quiet or slow-flowing bodies of freshwater [4,5,6]. Even though phytotoxicity studies on aquatic plants have traditionally been of minor importance in establishing regulatory measures concerning the environmental hazard of water contaminants (see [2,3]), duckweeds have a long history of being used to determine the toxic potential of water contaminants for aquatic higher plant life and to understand the toxicities involved [7]. The resurgence of interest in duckweeds as versatile model plants [8] helps consolidate the continuing importance of these macrophytes in the field of aquatic phytotoxicity.

Duckweeds are monocots that consist of flattened or rounded leaf- and thallus-like assimilatory organs or fronds that range from 1 to 15 mm in diameter or length and are only a few cells in thickness [5,8]. The fronds consist largely of spongy mesophyll with extensive air-filled intercellular spaces that confer buoyancy and some bear hairless adventitious root on the underside [5,8]. There are 35 species of duckweed [9,10] distributed among the five genera *Spirodela* (abbreviation *S.*), *Landoltia* (*La.*), *Lemna* (*Le.*), *Wolffiella* (*Wa.*) and *Wolffia* (*Wo.*), in addition to two interspecies hybrids [11,12,13,14] that exhibit genus-typical differences in the size and complexity of the fronds and in the number of roots they bear [5,6,8,15]; for pictorial representations, see [8,16,17]. Although they can flower and reproduce sexually, duckweeds grow mainly by vegetative propagation, with daughter fronds budding off from one or two meristematic pouches or pockets in the mother fronds [5,8]. This can take place very rapidly: duckweeds constitute the most rapidly growing higher plants [18,19]. The fronds remain temporarily attached to one another via connecting stalks or stipes, forming colonies of interconnected fronds that spread out over the water surface to ensure optimal access to water nutrients [8,20,21,22] that are taken up via the ventral frond surfaces and the roots (see [8]).

The rapid growth potential of duckweeds and their ability to take up numerous water-contaminating compounds in addition to nutrients have made these macrophytes promising agents for the remediation of industrial, agricultural and domestic wastewaters [8,16,17]. These attributes—combined with small size, simple structure and a consequent ease of cultivation and analysis—also facilitate the detection and interpretation of detrimental effects of water contaminants on aquatic higher plant material. Duckweeds accordingly are well-suited as model organisms for aquatic phytotoxicity testing in their amenability to the collection of data relevant to a variety of research questions, backed up by well-developed experimental protocols, a comprehensive literature base and an international expert community (see [23]). The International Steering Committee on Duckweed Research and Applications (ISCDRA) helps to guide, coordinate and communicate research on duckweeds, as detailed in the quarterly released Duckweed Forum newsletter that can be accessed via the website of the Rutgers Duckweed Stock Cooperative (RDSC; ruduckweed.org: see [8]).

## 2. Standardized Duckweed Toxicity Testing

Duckweeds have long been employed for testing for toxic effects of water-borne substances—often typical water contaminants—on higher plant life in aqueous environments: the pertinent literature up till 1990 was reviewed by Wang [7]. Based on these experiences, several national and international organisations concerned with the aquatic environment set up protocols for testing for water-borne toxicity to aqueous higher plants using either *Lemna minor* or *Lemna gibba* as model test organisms [16]: see Table 1.

These “classical” tests exemplified by the OECD Test No. 221 [24], the ISO test 20079 [25] and the USEPA test [26] summarized in [27] consist of the incubation of 9–12 fronds of a duckweed (*Le. minor* or *Le. gibba*: see [1] for photographic images) in at least 100 mL of mineral salt medium (SIS, 20xAAP or Steinberg) in correspondingly large vessels, such as beakers or culture dishes, both without and with several concentrations of a putatively toxic substance. After 7 days, growth parameters, including frond number, area and weight, are measured, and the chlorophyll content of the fronds can also be assessed. A more recent equivalent procedure has been prescribed by the Chinese agricultural industry standard NY/T 3090–2007 [28] (see [29]). An inhibition of frond growth and a reduction in the chlorophyll content of the cultures containing the test substance in comparison to the control define the toxicity of the test substance to the duckweed. Toxicity parameters can be calculated from dose-response curves for effective doses or concentrations (ECs) of test substances at effect levels such as 50%, 20% or 10% [30], e.g., EC_50_.

**Table 1:** National and international organisations that have developed standardized tests for waterborne toxicity to aquatic higher plants with duckweeds from 1990 to 2006. Bibliographic information for the standardized tests can be found in [27,31].

Organisation	Year	Duckweed	Country/area
ITM: Institute of Applied Environmental Research	1990	*Lemna minor*	Sweden
ASTM: American Society for Testing and Materials	1991	*Lemna gibba*	USA
APHA: American Public Health Association	1995	*Lemna gibba*	USA
SIS: Swedish Standards Institute	1995	*Lemna minor*	Sweden
AFNOR: Association Française de Normalisation	1996	*Lemna minor*	France
USEPA: US Environmental Protection Agency	1996	*Lemna* *minor*/*gibba*	USA
EC: Environment Canada	1999	*Lemna minor*	Canada
ISO: International Organisation for Standardization	2005	*Lemna minor*	International
OECD: Organisation for Economic Cooperation and Development	2006	*Lemna minor*/*gibba*	International

Duckweed growth proceeds exponentially over time under non-limiting growth conditions, since each frond replicates regularly. In this case, there is a linear relationship between frond number, area or weight (F), and time (t): the slope of the line represents the relative growth rate (RGR), which can be described as follows (see [18]):

RGR = lnF_t_ − lnxF_t0_/t − t0

It has been recommended that the doubling time of frond number in the control should be less than 2.5 days throughout the 7-day test period, which corresponds to an approximately 7-fold increase in frond number and a RGR of 0.275 d^−1^ [24], a criterion easily met by representative *Le. minor* and *Le. gibba* clones [18]. The RGR must remain constant throughout the incubation time of the assay to assure that assay conditions are not limiting the intrinsic growth rate of the duckweed. This means that—for the reliable assessment of phytotoxicity in terms of growth inhibition—the volume of medium, the nutrient content of the medium and the inoculum size must be designed to ensure that exponential growth can occur throughout the test period [32]. If the mineral salt content of the nutrient medium, for example, is not sufficient to maintain exponential growth of the control fronds throughout the assay duration, it might be sufficient to enable growth slowed by toxic influences to proceed in a continual exponential fashion that could thus not accurately be related to the control. The preliminary work required to ensure exponential control growth was important in designing the standard toxicity tests. According to the OECD, the necessary criteria for the validity of these assays can be attained using a static test regime, in which the test conditions at the start of the test are left without interference until the growth parameter measurements are made at the end of the 7-day incubation period [24], and this regime is usually adhered to.

An important factor for the comparability of toxicity data obtained with a standardized duckweed toxicity test over extended periods of time, including seasonal changes, is the stability of the intrinsic growth of the test organism. This stability ensures the re-usability of previously determined toxicity data in the context of new investigations. In a corresponding study, control growth data for toxicity tests with the same *S. polyrhiza* clone over a period of four years under identical test conditions revealed a good long-term stability of frond number and area parameters, although some variability with time and the season was noted [33].

The standardized tests outlined above are indeed well-suited for determining and defining overall toxicity to a duckweed because growth inhibition is indicative of global disturbances in the functioning of the test organism. This also applies to reduced chlorophyll synthesis—including the bleaching that often accompanies this—that reflects the loss of the photosynthetic potential that is the signature characteristic of most plant life. It is emphasized that the standardized tests are “toxicity tests” in the narrow sense of being concerned only with determining the deleterious potential *per se* of questionable substances on the test organism under defined, reproducible conditions in the laboratory. This sets them apart somewhat from “ecotoxicity testing”, which investigates the deleterious effects of water contaminants on duckweeds under conditions that are ecologically relevant, i.e., with respect to contaminant concentrations, the constitution of the medium and the surrounding conditions, and the duration and frequency of exposure [2]. Ecotoxicological testing with duckweeds makes use of the machinery of the “toxicity testing” discussed here to investigate the actual consequences of water pollution on aquatic higher plant life.

The standardized tests are relatively easy to carry out but are cumbersome due to the use of large culture vessels and culture medium volumes and are lengthy. Efforts to improve and complement the classical testing procedures are discussed in Section **3**. The standardized tests measure only overall growth-related endpoints and provide no information as to the course or the mechanism of the toxicity. However, the investigation of toxicity-related biomarkers for mechanistic interpretation of toxic effects has a long history of having been carried out parallel to the determination of toxicity itself. Section **4** deals with developments to improve the amount and quality of biomarker information obtainable for better mechanistic understanding of overall toxicity.

## 3. Determining Toxicity

One approach toward improving and extending the “classical” determination of the toxicity of water contaminants to duckweeds as laid down by the standardized toxicity tests has been largely concerned with efforts to miniaturize and speed up the standardized assay procedures with an eye to convenience and efficiency in carrying out large numbers of toxicity tests. This is discussed in the following Section 3.1. On the other hand, the possibility of using duckweed species other than *Le. minor* and *Le. gibba* as test organisms and the assessment of novel toxicity endpoints to good advantage has also been examined. These “alternative” developments are discussed in Section 3.2.

### 3.1. Modifying the Standardized Tests

Issues for improving the techniques of standardized duckweed assays proposed by the organizations shown in Table 1 for detecting toxicity have been the scaling down the of the assay dimensions, the shortening of the incubation period required to assess toxicity, ensuring the quality of the incubation medium during the duration of the test and the development of innovative procedures for growth parameter measurement and analysis.

#### 3.1.1. Downsizing and Timesaving

The standardized duckweed toxicity test assays recommend the use of relatively large culture vessels, medium volumes and duckweed innocula. The downsizing, or miniaturization, of the standard test format offers the advantages of reduced space requirements, lower costs, reduction in test solution volumes, less waste disposal and less starting plant material. Experimentation in this regard had already been carried out prior to the development of the standardized tests. Two reports from 1990 described a reduction in the solution volume for toxicity testing with *Le. minor* to 15 mL using 60 × 15 mm culture dishes and plastic cups [7,34]. Downsized versions of the standardized tests were later proposed in which five to six fronds of *Le. minor* are incubated in 10 mL of culture medium in six-well culture plates for 7 days [35,36]. The results obtained with tests of the toxicity of KCl and 3,5-dichlorophenol and of Zn nanoparticles in these miniaturized systems were reported to be comparable to those obtained with full-size standardized tests. A recent example of the use of this test format is the investigation of the toxicity of four antidepressants to *Le. minor* [37].

Further miniaturization had been reported as early as 2004, where two three-frond colonies of *Lemna paucicostata* (a synonym for *Lemna aequinoctialis*) were incubated for 7 days in 5 mL portions of medium containing numerous individual herbicides with different modes of action in 6-well culture plates [38], and two-frond colonies of *Le. gibba* were incubated for 7 days in 2.5 mL of test solution containing olive mill waste in the wells of 12-cell culture plates in 2007 [39]. Miniaturization has recently been described for the investigation of seven to eight fronds of *Le. minor* in 2 mL of medium containing various individual pesticides in the wells of 24-well culture plates [40]. It is questionable whether toxicity values determined with the formats of the two latter investigations indeed reflect the values that would be obtained with standardized test formats. The much higher frond number/medium volume ratios used in these studies in comparison with the standardized test formats may readily lead to frond crowding and nutrient limitation, which can quite generally be an issue in miniaturization. This would make the maintenance of exponential duckweed growth and a continuous RGR over the 7-day incubation period unlikely, which would—as discussed above—compromise the accuracy and reliability of the toxicity test. This has been indicated in an investigation in which the incubation of two fronds in 2 mL medium portions led to slower frond growth and deviant toxicity values in comparison with the results obtained in parallel tests with the standardized format [36] (see also the last paragraph of this section).

The recommended incubation period of the test substance with the duckweeds in the standardized assay procedures is 7 days. Although this exposure duration may actually be too short in terms of ecotoxicological testing [2], a shortening of the incubation period with the test substance can be advantageous for testing the toxicity of large numbers of samples in screening procedures and can also reduce the tendencies of frond crowding and nutrient limitation. Incubation times shorter than 7 days had already been employed prior to the establishment of the standardized tests (4 d in the case of *Le. minor* in 1986 (see [7]) but have seldom been used in the standardized formats since then. Two relatively recent examples are 4-day incubation periods in test procedures with *Landoltia punctata* in the presence of nanoparticular and soluble Cu [41] and with *Le. minor* and *Le. gibba* exposed to the pharmaceuticals chlorpromazine, paracetamol and diclofenac [42].

The miniaturization and incubation time reduction of the standardized assays can be combined. One recent example is the assessment of the phytotoxic effects of several environmentally relevant metals and metalloids to three to four fronds of *Le. gibba* incubated for three days in a test volume of 4 mL in 12-well culture plates by means of chlorophyll fluorescence imaging [43]. Growth and photosynthetic parameters were measured in the same samples. The inhibition of relative growth rates in terms of frond number and area were calculated from the obtained chlorophyll fluorescence images. Such multi-well, plate-based duckweed phytotoxicity assays significantly reduce the space, time and sample volume requirements of the standard duckweed growth inhibition tests. These benefits may, however, be compromised by lowered test sensitivity, with considerably higher EC_50_ values for growth inhibition being recorded in comparison with data obtained for standardized tests [43]. Nevertheless, multi-well plate-based duckweed phytotoxicity assays offer definite advantages in the rapid screening of large sample numbers. The benefits of miniaturization and short incubation times have become still more impressive, however, in the development of the duckweed phytotoxicity assays making use of alternative toxicity indictors to be discussed in Section 3.2.2.

#### 3.1.2. Ensuring the Quality of the Incubation Medium

Most toxicity tests with duckweeds proceed with incubation medium for duckweed growth containing the test substance being provided at the initiation of the test and not interfered with thereafter. The nutrients of the incubation medium in such “static” standard assays (already referred to in Section **2**) can become limiting during the 7-day test duration when the ratio of the inoculum to the incubation volume is too high and the incubation period is too long, especially in controls exhibiting vigorous growth. In addition, the concentration of test substances in the medium can also decrease significantly over long incubation periods due to take-up by the duckweed and especially when the test substances are unstable or volatile (see [44]). The initial composition of the medium (including the designated test substance concentration) can be approximately maintained when the incubation medium is periodically renewed and can be kept quite uniform in a flow-through setup. Protocols for employing medium renewal and flow-through in testing with *Le. minor* and *Le. gibba* have long been available (e.g., [45,46]), and the standardized assays allow for static, renewal and flow-through experimental formats (see, e.g., [24,33]).

A protocol based on the static ASTM test (see Table 1) for maintaining the quality of the medium in testing with *Le. gibba* was developed, according to which the medium containing the test substance is renewed daily [44]. This daily renewal ensures an approximately constant nutrient supply and test substance concentration and thus exposure to the test substance throughout the 7-day test period. However, such test procedures are considerably more time- and labour-intensive than static tests and have been seldom made use of. Flow-through setups to ensure continuous supply of nutrient medium are important for large-scale indoor cultivation of duckweeds (see [47]) but are too complicated and impractical to be of use in routine toxicity testing, especially where downsizing is advantageous.

#### 3.1.3. Data Imaging and Analysis

The simplest means of assessing duckweed growth in toxicity testing is via manual counting and weighing of the fronds. This was specified in the Environment Canada toxicity test (see Table 1) with *Le. minor* (see [31]) and is still sometimes employed to the present day, e.g., [48]. Further examples of manual frond counting have been cited [49], and the weighing of fronds must necessarily be carried out manually. The determination of frond area by means of digital image analysis has a long history: an early example of its use was in the assessment of dose-response relationships between 26 herbicides exhibiting 19 different modes of action on the growth of *Le. paucicostata* [38]. The OECD recommended photographic or digital imaging for recording frond area in its standardized assay procedure [24], which can be conveniently determined with free or commercially available image analysis software. Frond area determined by image analysis for the effects of metal pyrithione antifoulants on *Le. gibba* was found to be up to 10 times more sensitive than visually observed frond number as a toxicity index [50]. However, as indicated by the OECD [24], frond area determination must not necessarily derive from digital imaging: in a recent investigation of the toxicity of boron (B) to *Le. minor*, frond areas were calculated using the image analysis software Image J on photographic images of the fronds [51].

The investigation of 26 different herbicides on the growth of *Le. paucicostata* mentioned above [38] and an early application of the ISO 20079 protocol [25] to test the effect of heavy metals on *Le. minor* [30] used the LemnaTec Scanalyzer and LemnaTec SAW Lemna software (LemnaTec, Würselen, Germany) for the image capture and quantification of total frond area. The use of the LemnaTec Scanalyzer platform to calculate frond number and area in duckweed toxicity testing has been explained in detail [52] and used more recently, e.g., in tests of the effects of the chalcones salsolol A and B and the pseudogualanolide confertin on *Le. paucicostata* [53]. Other examples of image capture and analysis in duckweed toxicity testing are the use of the Nikon Digital Sight DS-5Mc camera with Nikon and Assess image analysis software to study the effect of olive mill wates on *Le. gibba* [39] and of the Canon EOS 450D digital camera and Image J analysis software in investigating the tolerance of *S. polyrhiza* and *Le. minor* to the pesticides atrazine and S-metalochlor [54]. Further software packages for processing digital frond images are the Image Tool analysis program used in the investigation of the toxicity of several antidepressants on *Le. minor* [37], the Lucia 5.0 program used to assess biogenic amine biosynthesis pathway changes associated with dye toxicity to *Le. minor* [55] and the Celleste 6 and Athena software programs marketed by ThermoFisher (Waltham, MA, USA) and IDEA Bio-Medical Ltd. (Rehovot, Israel). An automated microscopy approach to monitoring duckweed growth employing a Zeiss Axiocam 512 color CCD digital camera and Zen Blue 2.6 Professional software to capture fluorescence microscope images of *Le. minor* fronds that were processed by PlantCVv3.10.0 and OpenCVv3.4.9 analysis software may be adaptable for use in duckweed toxicity testing [56].

An interesting example is the use of the Zebrabox (ViewPoint Behavior Technology, Lyon, France), an automated observation and tracking system developed for zebrafish behaviour analysis, to study the growth inhibition potential of rare earth elements on *Wolffia globosa* [49]. Chlorophyll fluorescence detectors have long been used to assess chlorophyll content and chlorophyll fluorescence parameters in duckweed toxicity testing (see Section 4.2.1). The digital images used to determine chlorophyll fluorescence parameters can also be used to calculate the growth parameters of frond number and frond area, as shown in relation to metal and metalloid toxicity to *Le. gibba* [43]. This has the advantage of relating growth phenomena in terms of total frond area to photosynthetically competent leaf matter and the developmental status of the growing fronds [43,57]. A very recent new approach to quantifying duckweed growth in toxicity assessment is the quenching of the light emitted from an LED source by duckweed frond area coverage as registered by a solar cell connected to a digital multimeter. The greater the duckweed frond area produced by growth, the lesser the photoelectric signal emitted from the solar cell [58]. The use of this system was demonstrated with regard to the effects of the herbicides glyphosate and glufosinate and reportedly delivers reliable growth inhibition data within two days.

The optical biosensor system for the measurement of duckweed frond area is reported to be very cost-effective at USD 10 per unit [58]. Other low-cost technical approaches to digital imaging and analysis for objective and quantified biomonitoring have been proposed using standard digital cameras and Aphelion 3.02 framework software. These have been employed using a Nikon Coolpix 995 digital camera in the investigation of the toxicity of non-ionic detergents to *Le. minor* [59] and a Logitech HD Webcam C270 camera for method development with the reference substance potassium dichromate [60]. On a more sophisticated level, a machine learning-based tool (StarDist) simplifies and assists counting processes and helps arrange, manage and calculate inhibition percentages after duckweeds are exposed to contaminants. This was demonstrated in an investigation of the effects of herbicides and rare earths on *Wo. globosa* [49]. Duckbot, a machine customized for automating laboratory research workflows with duckweed, supports the setting up of sample plates with duckweed and growth media and gathering image data, as well as conducting relevant data analysis. Its use was demonstrated in a case study on the effect of salinity on the growth of *Sp. polyrhiza*, *Le. minor* and *Wolffia australiana* [61].

### 3.2. Alternatives to the Standardized Tests

Duckweed toxicity testing can vary fundamentally from the standardized procedures with regard to the test species used as toxicity vehicles and the endpoints determined to define toxicity. The use of these alternatives can also incorporate the downsizing and timesaving modifications of the standardized tests discussed above. Such tests can then be of value for high-volume toxicity testing if they can be shown to compare favourably with the standardized assay procedures.

#### 3.2.1. Alternative Test Organisms

Prior to the establishment of the standardized toxicity tests, a number of duckweed species, including *S. polyrhiza*, *Spirodela oligorhiza*, *La. punctata*, *Le. aequinoctialis*, *Lemna perpusilla* and *Lemna valdiviana*, had been used for investigating the effects of water pollutants on higher plants in addition to *Le. minor* and *Le. gibba* that were singled out as model organisms for the toxicity testing [7]. Some of these duckweeds have still been used occasionally as test organisms in toxicity tests. Employing *Le. aequinoctialis* for testing for water-borne toxicity [62,63] reflects the wish to use a tropical duckweed for use in tropical environments. *S. polyrhiza*, which has a long history as a test organism (see [64]), is used in both classical testing (e.g., [65]) and in newly developed tests using alternative endpoints (see Section 3.2.2). *La. punctata* [66,67] and *Wo. globosa* [49,68] are also used to test for water-borne toxicity on account of their importance in wastewater remediation. Most alternatively used duckweed species have no intrinsic advantages over *Le. minor* and *Le. gibba* as test organisms in traditional assay procedures, but *Wolffia* species would appear to be predestined for use in miniaturized and shortened versions of the standardized assays on account of their very small size and rapid growth.

#### 3.2.2. Alternative Indicators of Toxicity

The standard duckweed toxicity tests determine growth impairment in terms of the number, area, weight or chlorophyll content of whole, mature fronds. However, efforts have also been made to determine detrimental effects on root elongation and juvenile frond expansion that can indicate fundamental disturbances in growth—and hence toxicity—to the test organism. These alterative toxicity endpoints could be of value if they can be assessed as conveniently and accurately as frond growth in the standardized assays. Studies making use of such alternative indicators of toxicity are listed in Table 2.

Two of the alternative indices of toxicity presented in Table 2 constitute root length, either *per se* or upon re-growth after abscission. The three studies that measured the length of intact roots did not reveal any decisive advantage over the established standardized tests. They were limited to testing the effects of heavy metals on *Le. minor* and were based on the experimental methodology of the EC or OECD tests. They thus did not constitute any progress in the downsizing of these test procedures, and in only one instance [70] was a shorter incubation time of 4 days used. The measurement of the intact roots required for the tests is also laborious. However, in two instances [70,71], root length was judged to be a more sensitive and precise indicator of toxicity than frond number that was determined in parallel.

The promise of root length as an indicator of toxicity to duckweeds has been much better realized by the measurement of the re-growth of roots from fronds from which the roots were excised prior to the start of the experiment. This was shown in two pioneering studies in which the lengths of roots that re-grew after excision from *Le. minor*, *Le. gibba* and *Le. paucicostata* fronds were measured after exposure to several heavy metals [72] and to several herbicides [73]. These tests constituted drastic miniaturization and shortening of the standardized assay procedures in incubating single rootless fronds in 3 mL medium for 3 days in 24-well plates. They yielded E_c_ values for root re-growth with the investigated metals and herbicides comparable to those obtained for frond growth. This methodology was later systematically tested for its suitability as a generally applicable test procedure [27]. It was determined to be as sensitive as the conventional ISO 20079 test with the reference toxicant 3,5-dichlorophenol and was shown in national and international interlaboratory comparisons to be reliable and reproducible in tests with CuSO_4_ and wastewater. This validated *Lemna* root re-growth assay is thus simpler, more rapid, cheaper and more convenient (despite the dexterity required for the frond handling) than the standardized test procedures and is suitable for rapid screening of wastewater and natural waters. The fact that it is carried out with only one de-rooted frond per test for a short time indicates that the frond crowding and nutrient limitation that may be detrimental to the reliability of other miniaturized assays (see above) are probably not at issue here. The procedure was judged to be acceptable as a standardized biological test and for use as a regulatory tool and has accordingly now achieved the status of a standardized duckweed toxicity test in its own right (ISO 4979:2023 [79]). Recent studies have employed the root-re-growth assay as described here to investigate the effects of B on *Le. minor* [51] and of Cu and Cr on *S. polyrhiza* [75]. Despite the advantages of this miniaturized and rapid procedure, some very recent studies have investigated root re-growth upon exposure of *Le. minor* to cerium (Ce) [74] and microplastics [76] using the much more cumbersome methodology of the standardized OECD duckweed toxicity test.

An alternative toxicological indicator related to frond area rather than root length is the growth of the first fronds emerging from germinating turions of *S. polyrhiza*, which are overwintering derivatives of normal growing fronds that spend the winter at the bottom of water bodies in a dormant state and germinate to give rise to normal frond growth in the following spring (see [80]). A microbiotest featuring miniaturization, rapidity and convenience comparable to the root re-growth assays has been developed in which non-dormant *S. polyrhiza* turions are germinated and then incubated individually in 1 mL volumes of medium in 24-well culture plates for 3 days. A decrease in the area of the first frond that protrudes from the geminated turion, which can be digitally recorded and analyzed serves as a measure of toxicity [64]. Due to the investigation of only one germinated turion per test, frond crowding is not an issue and there should not be nutrient depletion sufficient to limit the growth of the first frond protruding from the turion. In addition to being simple and practical to perform, this test is largely independent of the maintenance of duckweed cultures, since the turion starting material can be readily stored until use. The test proved to be of comparable sensitivity to the standardized *Lemna* protocols in tests with 22 substances, including herbicides, organic and inorganic chemicals, and metals. Extensive international interlaboratory comparisons showed the test to be robust and reliable, and it was proposed to and accepted by the ISO as a standard toxicity test (ISO 20227:2017) for duckweeds [64,81]. The test is available as a patented commercial product (Duckweed Toxkit F; MicroBio Tests Inc., Gent, Belgium). It is—like the root re-growth assay—suitable for convenient high-throughput toxicity testing and is particularly useful for ecotoxicology work in the field. Other studies have also investigated the sensitivity of the germination and sprouting of *S. polyrhiza* turions to heavy metals (Ni, Cd and Cr; [77]) and determined that the single frond area produced by germinating turions was the most sensitive test endpoint for the toxic effect of Cd [78]. However, these experiments were carried out in formats resembling the original standardized test protocols and did not recommend systematic test procedures for routine testing. 

## 4. Describing Toxicity: Biomarkers of Toxic Effect

Although the standardized duckweed tests give information as to toxicity itself, it has always been of interest to know what causes the overall detrimental effect to the frond. Toxicity tests themselves have therefore long been accompanied by tests for biomarkers that can help to elucidate the mechanisms underlying the toxicity. Toxic substances that duckweeds can be exposed to include nutrients, metals and organic xenobiotics, the presence of which in natural waters is mostly, but not always, of anthropogenic origin. These substances elicit the generation of “biomarkers of toxic effect” when their influence is correlated with or causally linked to overall observed toxicity [1,82]. Toxicity tests with duckweeds coupled with biomarker-oriented analysis of the test organisms can reveal both the presence and the nature of water contaminant toxicity to aquatic higher plants. However, it should be noted that putatively toxic substance do not always elicit detectable biomarkers of toxic effect! For example, the treatment of *Le. minor* with several concentrations of perfluorooctanoic acid had no discernible effect on biometric frond parameters, chlorophyll content, photosynthetic electron rate or chlorophyll fluorescence parameters [83].

Biomarkers of toxic effect take many forms. Some expressions of toxic influence are readily evident as disturbances to structural and developmental features of duckweeds (see Section 4.1). The “classical” biomarkers of toxic effect include indicators of physiological aberrations of photosynthesis and respiration and are best known as biochemical and metabolic markers related to oxidative stress (Section 4.2). Techniques that are shaping modern biomarker analysis include indications of global alterations in DNA and polymeric structures, but most impressively feature extensive data generation through the application of the various “omics” that can lead to comprehensive understanding of mechanisms of toxicity (Section 4.3).

Biomarkers of toxic effect are in themselves often taken to be proof or verification of toxicity. This is especially evident when toxicity is discussed in terms of such biomarkers without any reference to an actual test of toxicity. An example is the analysis of assumed toxicity of silver (Ag) nanoparticles to *Wo. globosa* in terms of chlorophyll fluorescence parameters (even chlorophyll content is treated as a biomarker), oxidative damage metabolites and antioxidant enzymes [68]. Indeed, a case can be made for the idea that single or multiple biomarkers of effect can be regarded as direct evidence of toxicity, particularly when the effects identified by these biomarkers are obviously seriously detrimental to healthy duckweed growth. Biomarker data compiled during obviously toxic exposure may also correspond well with growth data obtained in the standardized toxicity assays. This was claimed, for example, for chlorophyll fluorescence parameters in comparing the toxicity of four herbicides to three *Lemna* species [73] (although a thorough later investigation of the effect of heavy metals on *Le. gibba* raised doubt about the accuracy of the correspondence [43]). However, biomarkers of effect are often specific for the water contaminant giving rise to them, and thus no particular biomarker can define a toxicity that is due to water contaminants in general, whereas growth inhibition is indeed a quite universal expression of toxicity.

It is preferable to regard the biomarkers being discussed here as being *descriptive* of toxicity, i.e., pointing to what is *going on* in toxicity. In contrast, the growth and chlorophyll synthesis impairment prescribed in the standardized tests are a measure of what has *gone on* in toxicity, i.e., what the result of the “goings-on” actually is. The observation of the biomarkers aims at explaining how the result in terms of growth inhibition came about. However useful, this approach to assessing biomarker data is not necessarily always strictly applicable, as biomarker alterations can take place in the absence of overt toxicity. An example is the detection of metabolite changes shown by ^1^H-NMR fingerprinting spectra without any macroscopic indication of toxicity in *Le. minor* exposed to several herbicides [84]. In such cases, biomarker changes can indicate impending toxicity at higher water-contaminant dosages.

### 4.1. Structural and Functional Aberrations

Some anatomical, morphological, developmental and physiological phenomena that may be readily observed in relation to toxicity testing are indicative of functional impairments in duckweeds due to exposure to water contaminants. An early finding was that exposure to heavy metals (Cd, Cu, Ni and Zn) leads to disturbances in the phototactic behaviour of *Le. minor* mesophyll cell chloroplasts [85]. Other aberrant responses of duckweeds under heavy metal stress summarized in [86] include frond chlorosis and developmental disturbances such as frond size reduction, frond abscission and colony disintegration [21,87,88], as well as root rejection and turion formation [89]. Indeed, standardized toxicity tests recommend the routine notation of such features to complement toxicity assessment (e.g., [24]). The floating up and first frond protrusion of germinating turions of *S. polyrhiza* were observed to be delayed in the presence of Cr, Cd and Ni ions [77,78]. A transition of the chloroplasts of *S. polyrhiza* and *Le. minor* fronds to chloro-amyloplasts and amylo-chloroplasts accompanied by a pronounced accumulation of transitory starch in the presence of Ni and Co was observed by electron microscopy [90,91]. These various phenomena all reflect toxic influences of metals as water contaminants rather than consummated toxicity itself.

### 4.2. “Classical” Biomarkers

Physiological, biochemical and metabolite biomarkers of toxic effect have long been determined in relation to water contaminant-induced toxicity to duckweeds. A review of biomarkers associated with toxic stress to duckweeds in addition to numerous other, primarily aquatic plants incorporates investigations dating from the end of the last century [82], and a more recent survey presents a comprehensive assessment of biomarkers of toxic effect specifically in duckweeds [1]. An overview of these biomarkers of toxic effect is presented in Table 3. The biomarkers constitute endpoints measured at the end of the incubation periods of toxicity assays. They have been determined in association with the standardized toxicity tests discussed in Section 2; there is as yet no comparable catalogue of biomarkers of toxic effect derived from the more recent standardized tests introduced in Section 3.2.2.

#### 4.2.1. Physiological, Biochemical and Metabolite Endpoint Biomarkers

An in-depth analysis of toxicity-related biomarkers is beyond the scope of this review (see [1,82] in this regard), but two complexes of biomarkers that are frequently documented are of particular interest. Many of the biomarkers listed in Table 3 are related to the wide-reaching toxic effects of oxidative stress that are frequently occasioned by exposure of duckweeds to water contaminants. This stress results from the production of reactive oxygen species (ROS), which represent the most important chemical biomarkers of toxic effect and lead to the production of metabolites such as the lipid peroxidation product MDA. In response, antioxidative solutes, such as glutathione and ascorbate, and antioxidative enzymes, such as peroxidase and catalase, are formed to counteract the effects of the ROS; Phase I and II enzymes are induced to detoxify water contaminants leading to oxidative stress (see [1,81] for detailed discussion). An interesting parallel to this elicitor–response scenario is the finding that biogenic amines—in particular, tyramine and spermine—accumulated significantly in *Le. minor* in response to toxic exposure to the antibiotic tetracycline [92]. A subsequent investigation illustrated the response of the biogenic amine biosynthesis pathway of the duckweed to toxic exposure to the dye gentian violet in terms of a stimulation of tyrosine decarboxylase (leading to tyramine accumulation) and inhibition of s-adenosylmethionine and ornithine decarboxylases [55]. Since the biomarkers under discussion here have been determined and compiled since the establishment of the standardized toxicity tests, they are not new in the sense of this review but rather represent the *status quo* of classical toxicity test results. They continue to be routinely determined up to the present: studies on the effects of norfloxacin on *S. polyrhiza* [65] and benzalkonium chloride on *Le. minor* [93] are just two recent examples.

Chlorophyll fluorescence also plays an important role as a “classical” biomarker of toxic effect in duckweeds. It is light re-emitted by light-excited chlorophyll molecules that return to non-excited states and is—as a non-invasive measurement of photosystem II (PSII) activity—an indicator of photosynthetic energy conversion in plants. Chlorophyll fluorometer protocols measure the photosynthetic efficiency of PSII, both in the light (ΔF/Fm’) and in a dark-adapted state (Fv/Fm) in terms of multiple fluorescence parameters (see [94]). Chorophyll fluorescence-based bioassays for rapid, high-throughput screening of toxic effects on the photosynthesis of aquatic macrophytes were introduced soon after the establishment of the standardized duckweed toxicity tests [95,96]. They have played an important role in duckweed toxicity testing ever since (see Table 3) and remain at the forefront of the characterization of toxic effects of water contaminants on duckweeds.

Chlorophyll fluorescence imaging-based phenotyping of *S. polyrhiza* exposed to Ni, Cr and NaCl showed that nine calculated fluorescence parameters exhibited very divergent sensitivities to dark- and light-adapted states of photosystem II activity, indicating the importance of assessing several chlorophyll fluorescence parameters in relation to toxicity [94]. This study also indicated that changes in duckweed photochemical efficiency do not necessarily correspond to growth responses. On the other hand, the exposure of *Le. minor* and *S. polyrhiza* to 3, 30 and 600 mg dimethyl phthalate (DMP)/L showed that multiple chlorophyll fluorescence parameters exhibited significant alteration only at the highest DMP concentration, in accordance with reduced chlorophyll accumulation as a standard toxicity index at this concentration only [97]. This study also highlighted the utility of leaf reflectance spectra, which reflect both leaf surface and internal structure and pigment characteristics, as biomarkers complementary to chlorophyll fluorescence parameters. The values of six spectral reflectance indices only varied significantly in concord with fluorescence parameter changes at the highest DPM concentration. In addition, the chlorophyll fluorescence imaging technique allowed the assessment of spatial variations in the distribution of chlorophyll fluorescence within the leaves. This has been further expanded upon by the use of chlorophyll fluorescence imaging to demonstrate the functional heterogeneity of fronds and colonies of *S. polyrhiza*, *La. punctata* and *Le. minor* treated with Ni and Cr [57].

#### 4.2.2. The Progression of Toxic Effects in Time

Most biomarkers of toxic effect are determined at defined times after the onset of treatment: at 1 to 7 days, as discussed above. Monitoring the appearance or development of such biomarkers within these timespans would permit judgement as to the progression of toxicity along a timescale. This can be important to judge whether standardized toxicological endpoint measurements reflect continual ongoing toxicity or if they result from toxic processes that are either belatedly or no longer effective.

Any non-invasive measurement regime leaving the test material intact, i.e., digital frond imaging with CCD cameras or chlorophyll fluorescence detectors, is suitable for repeated timeline measurements. An early example of this is the digital capture of *Le. paucicostata* frond images at daily intervals during the week-long exposure of the duckweed to numerous herbicides [38]. Visualization of the short-term progression of oxidative stress in *Le. minor* was described in 2007, whereby emissions of a fluorescent probe due to reactive oxygen species production elicited by the vitamin menadione were captured by an electron-multiplying CCD camera at intervals over a period of 6 h [98]. Although this was claimed to represent the observation of real-time toxicity, the continual CO_2_ uptake/emission measurements made with *Le. minor* exposed to four herbicides and four metals using a respiratory activity measuring system based on infrared (IR) technology over a 24 h period [99] more accurately reflect the progress of toxicity in real time. CO_2_ exchange rates were stimulated at low concentrations of the test substances and mostly inhibited at high concentrations. Short-term repetitive or continuous measurement of oxidative stress or CO_2_ exchange rates could accordingly be promising for initial screening for potential toxicity. Interestingly, the IR spectroscopy that was used to quantify decarboxylation reactions in biogenic amine biosynthesis [55] could also be used to monitor numerous toxicity-related enzyme reactions by determining absorbance changes in reactants over time [100,101].

Chlorophyll fluorescence parameters are most promising for repeated analysis in real time using the commercially available Imaging-PAM and FluorCam platforms (see [94]). Indeed, an Ecotox Photosystem Tool has recently been developed that combines the use of an Imaging PAM CCD camera and an IR gas analyser to enable simultaneous and continuous measurements and analysis of leaf area, CO_2_ exchange and chlorophyll fluorescence parameters [102]; see also [97]. Such a sophisticated system enables non-destructive analysis (allowing further investigation of the test material) that can detect multiple early toxicity symptoms even if no macroscopic indications of toxicity are evident.

### 4.3. Modern Methods of Biomarker Analysis

In recent years, sophisticated techniques for analysing transcripts, proteins and metabolites from plant material have made possible the determination of large numbers of biomarkers of toxic effect in duckweed tissues. These techniques can reveal numerous toxicity-associated biomarkers that are complementary to individual “classical” biomarkers that may have been detected. Such biomarkers constitute the “omics”, or the collective characterization and quantification of entire sets of biological molecules that translate into the structure, function and dynamics of an organism. The application of transcriptomics, proteomics, metabolomics and lipidomics to duckweed toxicity testing has greatly expanded the possibilities of achieving a comprehensive understanding of mechanisms underlying toxicity. This development has been complemented by the possibility of assessing changes in complex polymeric structures—including cellular DNA—that are associated with toxicity to duckweeds. Transformation has also become a tool in toxicity research in duckweeds, especially with respect to phytoremediation. Table 4 gives an overview of the investigations that have been carried out in these regards. The integration of the biomarker data available from these various modern approaches can throw comprehensive new light on toxicity to duckweeds.

#### 4.3.1. Transcriptomics

The microarrays that were in early use to detect differential gene expression in plant material were not made use of in duckweed toxicity testing, but modern transcriptomic analysis is now being applied in this regard. High-throughput or next-generation sequencing enables DNA and RNA to be sequenced in a rapid and cost-effective manner. It can reveal the differential gene expression that takes place in duckweeds in response to stimuli or triggers and leads to the overall response of the organism. It has been useful in this regard for understanding how duckweeds react and respond to stresses elicited by, e.g., ionizing radiation in *Le. minor* [116], salinity in *S. polyrhiza* [117], heat in *S. polyhiza* [118] and N starvation in *Le. aequinoctialis* [119]. It has also been used to analyse phytohormone-triggered developmental changes in duckweeds, e.g., salicylic acid-induced flowering in *Le. gibba* [120] and abscisic acid-induced turion formation in *S. polyrhiza* [121] (see also [122]). Transcriptomics has, however, only seldom been applied to the determination of toxicity due to water contaminants in duckweeds.

The inhibitory action of high concentrations of ammonium on the growth of *Le. minor* [123] was found to correspond to over 14,000 differentially expressed genes (DEGs) [103]. Most of the DEGs were downregulated under NH_4_^+^ toxicity. However, genes required for lignin biosynthesis in the phenylpropanoid biosynthesis pathway were upregulated, which indicates a strengthening of the cell wall. Genes related to programmed cell death were also upregulated, as were genes involved in the production of both reactive oxygen species (ROS) and the antioxidant enzyme system to scavenge the ROS.

The heavy metal cadmium (Cd) is a potent inhibitor of the growth of duckweeds such as *Le. minor* and *S. polyrhiza* [30,78]. A transcriptomic analysis of *La. punctata* to investigate the relative tolerance of this species to Cd [104] detected over 9000 DEGs associated with Cd toxicity, including genes associated with DNA repair and carbohydrate metabolic flux. Transcripts encoding enzymes involved in cell wall biosynthesis were downregulated, while the transcription levels of genes involved in glycolysis and the tricarboxylic acid cycle were upregulated. Upregulated transcripts encoded enzymes involved in sulphur assimilation and in antioxidation to combat ROS metabolism and led to enhanced expression of tonoplast-localized transporters to facilitate vacuolar accumulation of Cd. In a recent study of the tolerance of *S. polyrhiza* to heavy metal stress [105] (see also Section *4.5*), the expression of genes involved in fatty acid biosynthesis, β-oxidation and lipid degradation was shown to be upregulated to enable the incorporation of oxidative lipid degradation products into triacylglycerides to prevent cellular lipotoxicity due to the presence of unsaturated free fatty acids. A particularly highly expressed transcription factor was also indicated to be important in imparting heavy metal tolerance (see Section *4.5*).

Transcriptomics have also been employed in two recent studies examining the toxic effects of organic xenobiotics on duckweeds. In an examination of the influence of the pharmaceutical atorvastatin and the herbicide bentazon on *Le. minor* [106] (see also Section 4.3.2), the herbicide resulted in a far greater number of DEGs than did the pharmaceutical at low effective growth-inhibitory concentrations. Highly regulated genes of the ethylene-activated signalling pathway and of lipid and glycerophospholipid metabolism were differentially expressed only in response to atorvastatin, while the expression of highly regulated genes of cellular responses to light stimuli and to photosynthesis was affected only by exposure to bentazon, thus giving an indication of how specific the underlying mechanisms of toxicity can be with the elicitor. In an investigation of the mechanism of enantioselective toxicity, racemic- (Rac-), R- and S-preparations of the chiral imidazolinone pesticide imazamox (IM) were applied to *Le. minor* [29]. Transcriptional analysis showed that the treatments with Rac-, R- and S-IM produced 3107, 2580 and 13 DEGs, respectively, which were consistent with the comparative extent of toxicity of the three preparations to the duckweed. The DEGs resulting from Rac- and R-IM administration were related mainly to photosynthetic carbon assimilation and antenna proteins, glutathione metabolism, the pentose phosphate pathway, hormone signal transduction and zeatin biosynthesis. The DEGs in the samples treated with S-IM were specifically related to phenylalanine metabolism and phenylpropanoid biosynthesis. This study illustrates the specific toxicity and effects on transcription that enantiomers can exert.

Transcriptomic analyses of the effects of water contaminants of duckweeds are major projects that are not realistic options for routine and high-sample-volume toxicity testing. Their value for elucidating the toxicity mechanisms related to selected water contaminants, as illustrated above is, however, evident, and they should accompany the toxicity testing of as many substances as is practical for better understanding of both elicitor- and duckweed-specific toxicity. The comprehensive understanding of the gene expression involved in water-borne toxicity to duckweeds afforded by transcriptomic studies can be complemented by the use of further “omics” to illustrate the proteinaceous and metabolic consequences of the differential gene expression having taken place.

#### 4.3.2. Proteomics

Proteomics is a powerful tool for understanding changes in the plant proteome under toxic stress. Changes in the protein composition of duckweed fronds induced by exposure to water contaminants show the result of differential gene expression due to toxic influences. This can be determined by the identification of tryptic peptides of proteins extracted from control and stressed duckweed fronds by mass spectrometry and the quantitative relation of their spectra to known protein structures. Using this approach, an analysis of the effect of 15 days of exposure of *Le. minor* to aluminium (Al) detected 261 differentially expressed proteins (DEPs) identified by MS/MS parallel to growth inhibition and chlorosis of the fronds [107]. Most of the DEGs related to the citrate cycle and amino acid metabolism were upregulated, whereas those associated with energy metabolism and glyoxylate and dicarboxylate metabolism were predominantly downregulated. Antioxidant enzyme-related proteins were important in the response to Al.

A recent transcriptomic analysis of the toxic action of the pharmaceutical atorvastatin and the herbicide bentazon on *Le. minor* showed up to 1200 (80% downregulated) and 2700 DEGs (60% downregulated), respectively, depending on the elicitor concentrations employed [106] (see also Section 4.3.1). A complementary proteomic investigation showed that the regulation directions of the DEGs corresponded well to changes in the concentrations of the respective actual proteins, showing that DEGs can truly reflect changes in protein composition (and *vice versa*). An interesting aspect of this study was that toxicity was determined preliminarily over a period of 7 days with a standardized test, whereupon the sampling of the fronds for the transcriptomic and proteomic investigations was carried out after only three days of exposure to the toxins and thus during the development of the eventually determined toxicity.

#### 4.3.3. Metabolomics

Metabolomics is important for understanding the composition and changes in the plant metabolome under toxic stress. The metabolome of a plant at any one time represents the entirety of the small-molecule substrates, intermediates and products of cell metabolism present in the tissues, providing a direct functional readout of the physiological state of the plant material. Metabolomics can thus relate global changes in metabolite composition and changes in particular groups of metabolites to the detrimental impacts of water-borne substances on duckweeds revealed by toxicity tests. In an early attempt at this, ^1^H NMR fingerprints of extracts of *Le. minor* exposed to several different herbicides and a phytotoxin revealed elicitor-specific differences in metabolic composition due mainly due to differences in substances containing methylene, methine, hydroxy, amine, thiol, olefin and aldimine groups [84].

More recently, comprehensive metabolomic analyses of toxicity in duckweeds have been facilitated by the characterization of plant extracts by gas or liquid chromatography coupled with mass spectrometry. GC/EI/MS has been used to show that the application of the herbicides metribuzin and glyphosate and their mixtures to *Le. minor* results in an increase in the amino acid pool, thus indicating increased proteolytic activity, and in fluctuations in metabolites such as salicylate, caffeate, γ-aminobutyric acid, trehalose and squalene that indicate the activation of pathways involved in the relief of stress [108]. The anti-inflammatory drug diclofenac was shown by means of untargeted screening with RPLC-HILIC-ESI-TOF-MS to result in changes in organic acids, lignin, sugars, amino acids, dipeptides, flavonoids and fatty acids in *Lemna minor* that can be related to the activation of various defence mechanisms to ameliorate stress conditions. A targeted analysis of amino acid changes illustrated that different drug concentrations differentially influenced amino acid synthesis pathways [109].

The metabolome is quantitatively related to the other cellular ensembles of the genome, transcriptome, proteome and lipidome. The integration of metabolomics with other omics information can provide a better understanding of toxicity to duckweeds in the sense of systems biology. One recent attempt at this approach is the integration of transcriptomics and lipidomics (lipid-related metabolomics) in the investigation of the toxic effects of wastewater heavy metals on *S. polyrhiza* [105] (see also Section 4.3.1). This study revealed that the heavy metal treatment stimulated degradation of the mono- and digalactosyldiacylglyerol membrane lipids, resulting in the accumulation of toxic unsaturated free fatty acids, and that these harmful degradation products were removed by incorporation (and thus sequestration) into triacylglycerol. Parallel to these lipidomic findings, transcriptomic analysis revealed that genes involved in fatty acid synthesis, β-oxidation and lipid degradation were upregulated, while genes involved in cuticular wax biosynthesis were downregulated by the heavy metal treatment. The combination of metabolomics and transcriptomics led to a comprehensive understanding of the effects of heavy metals on the lipid metabolism of *Le. minor*. In the same vein, the complementation of transcriptomics by proteomics was important for the understanding of the influence of atorvastatin and bentazon on *Le. minor* referred to in Section 4.3.1 and Section 4.3.2 [106].

#### 4.3.4. FTIR Spectroscopy

Fourier-Transform Infrared Spectroscopy (FTIRS) of duckweed fronds exposed to toxic substances has enabled the detection of changes in complex polymeric molecules as well as in metabolites associated with toxicity. FTIRS revealed biochemical alterations attributable to structural protein, lipid, nucleic acid and carbohydrate changes associated with the toxicity of Cu, Cr, the herbicides atrazine and acetochlor, and heavy metal-containing industrial wastewaters to *Le. minor* [110]. In a similar vein, FTIRS showed that significant structural and functional alterations were induced in *Le. minor* at the biochemical level upon exposure to four per- and polyfluoroalkyl substances (PFASs) [111]. Even at PFAS concentrations that did not visibly inhibit growth, lipid peroxidation, protein aggregation, and protein and DNA conformation changes were observed: alterations in lipid, protein and DNA were concentration-related and compound-specific. This would make FTIRS a most sensitive indicator of even potential water-borne toxicity to duckweeds.

### 4.4. Genotoxicity

Are the toxic effects of water pollutants due merely to altered transcription and the consequential changes in enzyme activities and metabolite levels, or is damage to the DNA itself involved? While numerous environmental pollutants indeed cause damage to plant DNA [124], research on the impact of water contaminants on aquatic organisms has focused primarily on the physiological effects of the pollutants, while their genetic damage potential remains poorly understood [125]. The investigation of water-contaminant-related genotoxicity in duckweeds is still in its infancy.

DNA damage was indicated to accompany the effects of reduced photosynthetic pigment synthesis and increases in oxidative stress markers and antioxidant enzyme activities and metabolite concentrations in *Le. minor* exposed to mercury (Hg). The RAPD (random amplified polymorphic DNA) profile of the treated plants exhibited new bands and missing normal DNA amplicons, indicating a decline in genomic template stability [112]. The Comet assay that has been employed as a standard technique for evaluation of DNA damage/repair, biomonitoring and genotoxicity testing in animal research since the 1980s has only recently been used to detect DNA damage in both *Le. minor* and *S. polyrhiza* upon treatment with dimethyl phthalate (DMP) [114] and in *Le. minor* upon exposure to micro- and nano-zinc (Zn) particles [113]. The detection of the genotoxic effect of DMP as soon as 24 h after the onset of treatment indicates that DNA damage can provide a very rapid indication of severe toxic effects. The DNA damage was considered in both studies to result from the impact of reactive oxygen species (ROS) formed as a response to the DMP and Zn particle treatments, in agreement with the view that DNA damage quite generally results from oxidative stress [124]. The routine investigation of potential genotoxicity could be profitably integrated into toxicity testing with duckweeds to provide a comprehensive overview of the damage to DNA that water contaminants can exert on duckweeds as representatives of higher aquatic plants. This could be most practically realized by extensive use of the widely used Comet assay that is a versatile, sensitive and relatively straightforward procedure that allows for high-throughput screening [125]. Since the Comet assay indicates reversible DNA damage [125], it provides the opportunity to better understand metabolic responses to water-borne toxicants in terms of how these responses (e.g., the enhancement of antioxidative solute concentrations and enzyme activities) may relate to the alleviation of conditions leading to DNA damage (i.e., the presence of high reactive oxygen species contents) to allow DNA repair to take place.

### 4.5. Transformation

The diagnostic value of biomarkers identified in relation to toxicities to duckweeds may be enhanced in some cases by integrating transformation procedures into the analysis of toxicity. This has recently been the case in two examinations of heavy metal toxicity primarily concerned with understanding and improving the tolerance of duckweeds to heavy metals. Tolerance to heavy metals—or other water contaminants—is an important property of duckweeds in their use in the remediation of contaminated wastewater or natural waters, which is an important opportunity for putting duckweeds to good use [16,17,126]. All manner of water contaminants can be removed from water by duckweeds, the versatility of *Le. minor* in this regard being particularly impressive [127]. A typical recent environmentally relevant example illustrates the potential of a duckweed (*Le. aequinoctialis*) to remove contaminants (the metals Cd, Cr, Pb and V) from artificial wetland water [128]. The remediative ability of duckweeds presupposes the ability of the macrophytes to tolerate certain levels of the toxicants that are to be taken up: increased tolerance enhances the remediative potential.

In one case, the biomarker providing an impetus for the transformation was a metabolite, in the other a gene expression product. It is of note that neither of these studies employed a toxicity test per se to document the heavy metal toxicity underlying tolerance phenomena (although some indications of toxicity compatible with such tests were provided): they illustrate the use of biomarkers to define toxicity (see Section **4**).

One of the metabolites found to respond to Cd stress in *Lemna turionifera* (indicated morphologically by increased root abscission) is glutamate (Glu), the content of which increased and activated the Ca^2+^ signalling pathway in fronds [129]. Phosphoserine aminotransferase (*PSAT*), a crucial enzyme in Glu metabolism, from *Arabidopsis thaliana* was introduced into *Le. turionifera*, and the response of the transformed duckweed to exposure to Cd was examined [115]. The *PSAT*-transgenic duckweed proved to be better-suited to tolerate Cd stress than the wild type, as indicated by prevention of root abscission, increased photosynthetic capacity and pigment content, and upregulated antioxidant enzyme activities. In addition, an upregulation of Glu metabolism-related enzymes and an increased Glu content were determined by transcriptomic and metabolomic analyses. The observation of Glu as a Cd toxicity-related biomarker thus led to the insight of the probable importance of Glu metabolism for heavy metal tolerance in aquatic plants.

A transformational offshoot of the transcriptomic identification of a biomarker was a valuable component of a study providing insight into the mechanisms of heavy metal stress in *S. polyrhiza* [105]. Treatment of the duckweed with heavy metals in flue gas desulphurization (FGD) wastewater resulted in reduced growth, chlorosis and decreased chlorophyll content of the fronds, which are indeed criteria of toxicity according to the standardized duckweed toxicity assays. Lipidomic and transcriptomic investigations revealed reduced membrane lipid biosynthesis and stimulated lipid degradation resulting in triacylglycerol and carbohydrate accumulation. Fatty acid biosynthesis, β-oxidation and lipid degradation-related genes were upregulated, in contrast to downregulated cuticular wax biosynthesis genes. Of particular interest was the identification of a highly upregulated transcription factor gene (SpWRI3), the ecotopic expression of which in *A. thaliana* led to increased tolerance of the transgenic plant to FGD wastewater (lower malondialdehyde and higher glutathione levels). This indicates that SpWRI3 is important for the tolerance of *S. polyrhiza* to heavy metals.

The insights into water contaminant tolerance gained from these studies suggest that targeted transformation of duckweeds, for which protocols have been developed (see [8,126]), can be an effective tool to improve the water remediation capacity of the macrophytes. An example is the overexpression of the photorespiratory pathway gene serine: glyoxylate aminotransferase from *A. thaliana* that led to salt stress tolerance in *Le. minor* [130]. Transformation can be a useful tool for understanding toxic scenarios, as illustrated in the two examples detailed above, but the advisability of its actual implementation can only be decided upon on the basis of appropriate toxicity indices on an individual basis. It is not suitable for routine introduction into duckweed toxicity testing, as it does not reveal the results of toxicity itself but is rather a specifically defined genetic manipulation useful for the interpretation, and possibly the exploitation, of these results. Transformation is not the only molecular tool that can be used to this end. The genetic constitution of duckweeds may also be able to be altered to improve the water-remediative attributes of the macrophytes by techniques such as the artificial miRNA gene silencing shown to suppress the expression of the magnesium chelatase subunit *CH42* that resulted in a reduction in chlorophyll pigmentation in *Le. minor* [131]. Another promising technique is the CRISPR/Cas9-mediated gene editing [132] that produced biallelic mutations with an albino phenotype in *Le. aequinoctialis* [133] in its only application to date in duckweeds.

## 5. Conclusions

The standardized tests that were established between 1991 and 2006 remain popular to the present and are still quite adequate for determining water-borne toxicity to duckweeds as representatives of higher aquatic plant life. Experience has shown that duckweed species other than *Le. minor* and *Le. gibba*—especially *S. polyrhiza*—are also suitable for toxicity testing. Efforts to downsize and shorten the test procedures have been most successful with the development of new standardized toxicity tests using the endpoints of root re-growth and first-frond growth immediately following turion germination. The classical metabolic, enzymatic and physiological biomarkers that have long been determined in the context of toxicity to duckweeds are still valuable descriptors of water-borne toxicity, and progress has been made in monitoring them along time-lines. However, recent developments in transcriptomics, proteomics and metabolomics—complemented by genotoxicity assays and transformation approaches—can provide much more toxicity-related data that are invaluable for the understanding of the mechanisms underlying toxicity. In an earlier paper, a proposal was made for the identification of unknown toxicants in contaminated water samples on the basis of toxicant-biomarker databases [1]. The realization of this goal becomes ever more feasible with the wealth of biomarkers of toxic effect that can be made available by the modern approaches to duckweed toxicity description described in this review.

## Figures and Tables

**Table 2:** Toxicity indicators in tests deviating from the growth parameters used in the standardized toxicity tests listed in Table 1.

Toxicity indicator	Toxin	Duckweed species	Reference
Decrease in root length	Ni	*Lemna minor*	[69]
	Pb	*Lemna minor*	[70]
	Cd	*Lemna minor*	[71]
Impaired re-growth of roots after excision	Ag, Cd, Cr, Cu, Hg	*Lemna* *minor/gibba/paucicostata*	[72]
	atrazine, diuron, paraquat, simazine	*Lemna* *minor/gibba/paucicostata*	[73]
	Cu	*Lemna minor*	[27]
	Ce	*Lemna minor*	[74]
	B	*Lemna minor*	[51]
	Cu, Cr	*Spirodela polyrhiza*	[75]
	microplastic	*Lemna minor*	[76]
Inhibited growth of first frond produced by germinating turions	22 substances: herbicides, organic/inorganic compounds, metals	*Spirodela polyrhiza*	[64]
	Ni, Cd, Cr	*Spirodela polyrhiza*	[77,78]

**Table 3:** Examples of “classical” biomarkers determined to accompany toxicity to duckweeds exposed to various metals and organic xenobiotics. The “examples” and “duckweed species tested” are assigned to the respective water contaminants; the right-hand column summarily lists the biomarkers determined in such investigations. Content taken from [1,82], in which the data summarized here are comprehensively presented and discussed. MDA: malondialdehyde, SOD: superoxide dismutase.

Water contaminant	Examples	Duckweed species tested	Biomarkers
**Metals**			altered photosynthesis pigment contents/ratios perturbations of chloro-plast ultrastructure altered chlorophyll fluorescence parameters impaired photosynthe-sis photochemistry inhibited photosyn-thetic O_2_-evolution increased reactive oxygen species (ROS) content increase in lipid peroxidation products (MDA) increased Phase I enzyme (functionali-zing: e.g., peroxidases, esterase) activities increased Phase II enzyme (conjugating: e.g., glutathione-S-transferase, methyl-transferase) activities increased antioxidant enzyme (e.g., peroxi-deses, catalase, SOD) activities increased antioxidant solute (e.g., ascorbate, glutathione, thiols) concentrations increased contents of phenols, sterols and flavonoids increases in starch synthesis enzyme activities starch accumulation
Heavy metals	Cd, Co, Cu, Cr, Hg, Zn, Ni, Pb	*S. polyrhiza* *La. punctata* *Le. minor*	
Nanoparticles	Ag, Cu	*S. polyrhiza* *Le. minor* *Wo. globsa*	
Metalloids	As, B, Se	*La. punctata* *Le. minor/gibba* *Wo. arrhiza*	
Rare earths	Pr, Ce	*S. polyrhiza*	
**Organic xenobiotics**			
Natural chemicals: * bacterial toxins* * coal tar hydrocarbons*	coronatine fluoranthine	*Le. paucicostata* *Le. minor*	
Industrial chemicals: *solvents* *dyes* *surfactants*	diethyl phthalate Malachite Green alkyl dimethyl amine oxide	*S. polyrhiza* *Le. minor* *Le. minor*	
Agricultural chemicals: * herbicides* * insectucides* * fungicides* * growth retardants* * anti-ozonants*	atrazine teflubenzuron Epoxiconazole uniconazole ethylene diurea	*Le. minor* *Le. minor* *Le. minor* *La. punctata* *Le. minor*	
Pharmaceuticals: * analgesics* * antibiotics* * anti-depressants*	diclophenac tetracycline fluoxetine	*Le. minor* *Le. minor* *Le. minor*	
Particles:	polyethylene microbeads	*Le. minor*	

**Table 4:** Investigations carried out to describe toxicity to duckweeds in contemporary approaches complementary to the acquisition of the “classical” biomarkers of effect referred to in Section *4.2*. The results of the individual studies are discussed in the text.

Type of investigation	Toxin	Duckweed species	Reference
**Transcriptomics**	NH_4_^+^	*Lemna minor*	[103]
	Cd	*Landoltia punctata*	[104]
	heavy metal mixture	*Spirodela polyrhiza*	[105]
	atorvastatin/bentazon	*Lemna minor*	[106]
	imazamox	*Lemna minor*	[29]
**Proteomics**	Al	*Lemna minor*	[107]
	atorvastatin/bentazon	*Lemna minor*	[106]
**Metabolomics**	herbicides, phytotoxin	*Lemna minor*	[84]
	herbicides	*Lemna minor*	[108]
	diclofenac	*Lemna minor*	[109]
**Lipidomics**	heavy metal mixture	*Spirodela polyrhiza*	[105]
**FTIR spectroscpy**	herbicides, heavy metals	*Lemna minor*	[110]
	fluoralkyls	*Lemna minor*	[111]
**Genotoxicity**	Hg	*Lemna minor*	[112]
	Zn-MP/NP	*Lemna minor*	[113]
	dimethyl phthalate	*Spirodela polyrhiza*	[114]
**Transformation**	Cd	*Lemna turionifera*	[115]
	heavy metal mixture	*Spirodela polyrhiza*	[105]

## Data Availability

No new data were created or analyzed in this study.

## References

[1] Ziegler P., Sree K.S., Appenroth K.-J. (2018). Duckweed biomarkers for identifying toxic water contaminants?. Environ. Sci. Pollut. Res..

[2] Ceschin S., Bellini A., Scalici M. (2021). Aquatic plants and ecotoxicological assessment in freshwater ecosystems: A review. Environ. Sci. Pollut. Res..

[3] Lewis M.A. (1995). Use of freshwater plants for phytotoxicity testing: A review. Environ. Pollut..

[4] Sculthorpe C.D. (1967). The Biology of Aquatic Vascular Plants.

[5] Landolt E. (1986). The Family of Lemnaceae—A Monographic Study, Vol. 1. Biosystematic Investigations in the Family of Duckweeds (Lemnaceae) (Vol. 2).

[6] Bog M., Appenroth K.-J., Sree K.S. (2020). Key to the determination of taxa of Lemnaceae: An update. Nord. J. Bot..

[7] Wang W. (1990). Literature review on duckweed toxicity testing. Environ. Res..

[8] Acosta K., Appenroth K.J., Borisjuk L., Edelman M., Heinig U., Jansen M.A.K., Oyama T., Pasaribu B., Schubert I., Sorrels S. (2021). Return of the Lemnaceae: Duckweed as a model plant system in the genomics and postgenomics era. Plant Cell.

[9] Bog M., Appenroth K.-J., Sree K.S. (2019). Duckweed (Lemnaceae): Its molecular taxonomy. Front. Sustain. Food Syst..

[10] Bog M., Sree K.S., Fuchs J., Hoang P.T.N., Schubert I., Kuever J., Rabenstein A., Paolacci S., Jansen M.A.K., Appenroth K.-J. (2020). A taxonomic revision of *Lemna* sect. Uninerves (Lemnaceae). Taxon.

[11] Braglia L., Lauria M., Appenroth K.-J., Bog M., Breviario D., Grasso A., Gavazzi F., Morell L. (2021). Duckweed species genotyping and interspecific hybrid discovery by tubulin-based polymorphism fingerprinting. Front. Plant Sci..

[12] Braglia L., Ceschin S., Iannelli M.A., Bog M., Fabriani M., Frugis G., Gavazzi F., Giani S., Mariani F., Muzzi M. (2024). Characterization of the cryptic interspecific hybrid *Lemna* × *mediterranea* by an integrated approach provides new insights into duckweed diversity. J. Exp. Bot..

[13] Appenroth K.-J., Jansen M.A.K., Lam E., Shoham T., Sree K.S. (2024). Important terms in duckweed research defined. Duckweed Forum (ISCDRA Newsl.).

[14] Romano L.E., Braglia L., Iannelli M.A., Lee Y., Morello L. (2024). A survey of duckweed species in southern Italy provided first occurrences of the hybrid *Lemna* × *mediterranea* in the wild. bioRxiv preprint.

[15] Tippery N.P., Les D.H., Cao X., Fourounjian P., Wang W. (2020). Tiny plants with enormous potential: Phylogeny and evolution of duckweeds. The Duckweed Genomes.

[16] Ziegler P., Sree K.S., Appenroth K.-J. (2016). Duckweeds for water remediation and toxicity testing. Toxicol. Environ. Chem..

[17] Ziegler P., Sree K.S., Appenroth K.-J. (2017). The uses of duckweed in relation to water remediation. Desalin. Water Treat..

[18] Ziegler P., Adelmann K., Zimmer S., Schmidt C., Appenroth K.-J. (2015). Relative in vitro growth rates of duckweeds (Lemnaceae) ─ The most rapidly growing higher plants. Plant Biol..

[19] Sree K.S., Sudakaran S., Appenroth K.-J. (2015). How fast can angiosperms grow? Species and clonal diversity of growth rates in the genus *Wollfia* (Lemnaceae). Acta Physiol. Plant..

[20] Kandeler R. (1988). Überlebensstrategien bei Wasserlinsen. Biol. Rundsch..

[21] Topp C., Henke R., Keresztes A., Fischer W., Eberius M., Appenroth K.J. (2011). A novel mechanism of abscission in fronds of *Lemna minor* L. and the effect of silver ions. Plant Biol..

[22] Kim I. (2016). Structural differentiation of the connective stalk in *Spirodela polyrhiza* (L.) Schleiden. Appl. Microsc..

[23] Laird R.A., Barks P.M. (2018). Skimming the surface: Duckweed as a model system in ecology and evolution. Am. J. Bot..

[24] OECD (Organisation for Economic Co-operation and Development) (2006). Test No. 221: *Lemna* sp. Growth Inhibition Test. OECD Guidelines for the Testing of Chemicals.

[25] (2005). Water Quality—Determination of the Toxic Effect of Water Constituents and Wastewater on Duckweed (Lemna minor)—Duckweed Growth Inhibition Test.

[26] USEPA (United States Environmental Protection Agency) (2012). Aquatic Plant Toxicity Test Using Lemna spp. Ecological Effects Test Guidelines OPPTS 850.4400.

[27] Park J., Yoo E.-J., Shin K., Depuydt S., Li W., Appenroth K.-J., Lillicrap A.D., Xie L., Lee H., Kim G. (2022). Interlaboratory validation of toxicity testing using the duckweed *Lemna minor* root-regrowth test. Biology.

[28] (2017). Chemical Pesticide-Guideline for *Lemma* sp. Growth Inhibition Test. Chinese Agriculture Industry Standard B17, Ministry of Agriculture Bulletin No. 2540.

[29] Li R., Luo C., Qiu J., Li Y., Zhang H., Tan H. (2022). Metabolomic and transcriptomic investigation of the mechanism involved in enantioselective toxicity of imazamox in *Lemna minor*. J. Hazard. Mater..

[30] Naumann B., Eberius M., Appenroth K.-J. (2007). Growth rate based dose-response relationships and EC-values of ten heavy metals using the duckweed growth inhibition test (ISO 20079) with *Lemna minor* L.. clone St. J. Plant Physiol..

[31] EC Biological Test Method (2007). Test for Measuring the Inhibition of Growth Using the Freshwater Macrophyte, Lemna minor: Report EPS 1/RM/37.

[32] Huebert D.B., Shay J.M. (1993). Considerations in the assessment of toxicity using duckweeds. Environ. Toxicol. Chem..

[33] Olah V., Hepp A., Vaca N.Y.G., Tamas M., Meszaros I. (2018). Retrospective analyses of archive phytotoxicity test data can help in assessing internal dynamics and stability of growth in laboratory duckweed cultures. Aquat. Toxicol..

[34] Taraldsen J.E., Norberg-King T.J. (1990). New method for determining effluent toxicity using duckweed (*Lemna Minor*). Environ. Toxicol. Chem..

[35] Soukupova I., Beklova M. (2010). Validation of duckweed microbiological test for assessing hazardous substances. J. Biochem. Technol..

[36] Kalcikova G., Marolt G., Kokali A.J., Gorvajn A.Z. (2018). The use of multiwell culture plates in the duckweed toxicity test—A case study on Zn nanoparticles. New Biotechnol..

[37] Drobniewska A., Giebultowicz J., Wawryniuk M., Kierczak P., Nalecz-Jawecki G. (2024). Toxicity and bioaccumulation of selected antidepressants in *Lemna minor* (L.). Ecohydrol. Hydrobiol..

[38] Michel A., Johnson R.D., Duke S.O., Scheffler B.E. (2004). Dose-response relationships between herbicides with different modes of action and growth of *Lemna paucicostata*: An improved ecotoxicological method. Environ. Toxicol. Chem..

[39] Cayuela M.L., Millner P., Slovin J., Roig A. (2007). Duckweed (*Lemna gibba*) growth inhibition bioassay for evaluating the toxicity of olive mill wastes before and during composting. Chemosphere.

[40] Rivenbark K.J., Nikkhah H., Wang M., Beykal B., Phillips T.D. (2024). Toxicity of representative organophosphate, organochlorine, phenylurea, dinitroaniline, carbamate, and viologen pesticides to the growth and survival of *H. vulgaris*, *L. minor*, and C. elegans. Environ. Sci. Pollut. Res..

[41] Shi J., Abid A.D., Kennedy I.A., Htristova K.R., Silk W.K. (2011). To duckweeds (*Landoltia punctata*), nanoparticulate copper oxide is more inhibitory than the soluble copper in the bulk solution. Environ. Pollut..

[42] Alkimin G.D., Daniel D., Frankenbach S., Serodio J., Soares A.M.V.M., Barata C., Nunes B. (2019). Evaluation of pharmaceutical toxic effects of non-standard endpoints on the macrophyte species *Lemna minor* and *Lemna gibba*. Sci. Total Environ..

[43] Irfan M., Meszaros I., Szabo S., Olah V. (2024). Comparative phytotoxicity of metallic elements on duckweed *Lemna gibba* L. using growth- and chlorophyll fluorescence induction-based endpoints. Plants.

[44] Brain R.A., Solomon K.R. (2007). A protocol for conducting 7-day daily renewal tests with *Lemna gibba*. Nat. Protoc..

[45] Walbridge C.T. (1977). A Flow-Through Testing Procedure with Duckweed (Lemna minor L.).

[46] Davis J.A. (1981). Comparison of Static-Replacement and Flow-Through Bioassays Using Duckweed.

[47] Coughlan N.E., Walsh E., Bolger P., Burnell G., O’Leary N., O’Mahoney M., Paolacci S., Wall D., Jansen M.A.K. (2022). Duckweed bioreactors: Challenges and opportunities for large-scale indoor cultivation of Lemnaceae. J. Clean. Prod..

[48] Wilson P.C., Hinz F.O., Farrell I. (2024). Impacts of fulvic acid on the toxicity of the herbicide atrazine to *Lemna minor*. Bull. Environ. Contam. Toxicol..

[49] Kurnia K., Lin Y.-T., Farhan A., Malhotra N., Luong C.T., Hung C.-H., Roldan M.J.M., Tsao C.-C., Cheng T.-S., Hsiao C.-D. (2023). Deep learning-based automatic duckweed counting using StarDist and its application on measuring growth inhibition potential of rare earth elements as contaminants of emerging concern. Toxics.

[50] Okamura H., Togosmaa L., Sawamoto T., Fukushi K., Nishida T., Beppu T. (2012). Effects of metal pyrithione antifoulants on freshwater macrophyte *Lemna gibba* G3 determined by image analysis. Ecotoxicology.

[51] Cui R., Kwak J.I., An Y.-J. (2024). Understanding boron toxicity in aquatic plants (*Salvinia natans* and *Lemna minor*) in the presence and absence of EDTA. Aquat. Toxicol..

[52] Jansen M., Nagel K. (2016). Useful methods (6): LemnaTec Scanalyzer, a useful tool for duckweed research and testing. Duckweed Forum.

[53] Perera W.H., Meepagala K.M., Fronczek F.R., Cook D.D., Wedge D.E., Duke S.O. (2019). Bioassay-guided isolation and structure elucidation of fungal and herbicidal compounds from *Ambrosia Salsola* (Asteraceae). Molecules.

[54] Cruz F.V.d.S., Brant H.S.C., Ohlund L., Sleno L., Juneau P. (2024). Tolerance and phytoremediation capacity of atrazine and S-metaloclor by two duckweeds. Environ. Sci. Pollut. Res..

[55] Adomas B., Sikorski L., Bes A., Warminski K. (2020). Exposure of *Lemna minor* L. to gentian violet or Congo red is associated with changes in the biosynthesis pathway of biogenic amines. Chemosphere.

[56] Cox K.L., Manchego J., Meyers B.C., Czymmek K.J., Harkness A. (2022). Automated imaging of duckweed growth and development. Plant Direct.

[57] Olah V., Kosztanko K., Irfan M., Szabo S.B., Jansen M.A.K., Szabo S., Meszaros I. (2024). Frond-level analyses reveal functional heterogeneity within heavy metal-treated duckweed colonies. Plant Stress.

[58] Lai Y.-J., Lu P.-C., Kung Y. (2025). Duckweed-based optical biosensor for herbicide toxicity assessment. Biosens. Bioelectron..

[59] Mazur R., Szoszkiewicz K., Lewicki P., Bedla D. (2018). The use of computer image analysis in a *Lemna minor* L. bioassay. Hydrobiologia.

[60] Haffner O., Kucera E., Drahos P., Ciganek J., Kozakova A., Urminska B. (2020). *Lemna minor* bioassay evaluation using computer image analysis. Water.

[61] Subbaraman B., de Lange O., Ferguson S., Peek N. (2024). The Duckbot: A system for automated imaging and manipulation of duckweed. PLoS ONE.

[62] Bengtsson B.-E., Bongo J.P., Eklund B. (1999). Assessment of duckweed *Lemna aequinoctialis* as a toxicological bioassay for tropical environments in developing countries. Ambio.

[63] Trenfield M.A., Harford A.J., Mooney T., Ellis M., Humphrey C., van Dam R.A. (2019). 2019. Integrating laboratory and field studies to assess impacts of discharge from a uranium mine and validate a water quality guideline value for magnesium. Integ. Environ. Assess. Manag..

[64] Baudo R., Foudoulakis M., Arapi G., Perdaen K., Lanneau W., Paxinou A.-C.M., Kouvdou S., Persoone G. (2015). History and sensitivity comparison of the *Spirodela polyrhiza* microbiotest and *Lemna* toxicity tests. Knowl. Manag. Aquat. Ecosyst..

[65] Zhao X.-L., Li P., Qu C., Lu R., Li Z.-H. (2022). Phytotoxicity of environmental norfloxacin concentrations on the aquatic plant *Spirodela polyrrhiza*: Evaluation of growth parameters, photosynthetic toxicity and biochemical traits. Compar. Biochem. Physiol. Part C.

[66] Lalau C.M., Simioni C., Vicentini D., Ouriques L.C., Mohedano R.A., Puerari R.C., Matias G. (2020). Toxicological effects of AgNPs on duckweed (*Landoltia punctata*). Sci. Total Environ..

[67] Yang G.-L., Huang M.J., Tan A.-J., Lv S.-M. (2021). 2021. Joint effects of naphthalene and microcystin-LR on physiological responses and toxin bioaccumulation of *Landoltia punctata*. Aquat. Toxicol..

[68] Zou X., Li P., Huang Q., Zhang H. (2016). The different response mechanisms of *Wolffia globosa*: Light-induce silver nanoparticle toxicity. Aquat. Toxicol..

[69] Antunes P.M.C., Kreager N.J. (2014). Lead toxicity to *Lemna minor* predicted using a metal speciation chemistry approach. Environ. Toxicol. Chem..

[70] Gopalapillai Y., Vigneault B., Halo B.A. (2014). Root length of aquatic plant *Lemna minor* L. as an optimal toxicity endpoint for biomonitoring of mining effluents. IEAM (Integr. Environ. Assess. Manag.).

[71] Xue Y., Wang J.-q., Huang J., Li F.-y., Wang M. (2018). The response of duckweed (*Lemna minor* L.) roots to Cd and its chemical forms. J. Chem..

[72] Park A., Kim Y.-J., Choi E.-M., Brown M.T., Han T. (2013). A novel bioassay using root re-growth in *Lemna*. Aquat. Toxicol..

[73] Park J., Brown M.T., Depuydt S., Kim J.K., Won D.-S., Han T. (2017). Comparing the acute sensitivity of growth and photosynthetic endpoints in three *Lemna* species exposed to four herbicides. Environ. Pollut..

[74] Liu Y., Zhao X., Ma Y., Dai W., Song Z., Wang Y., Shen J., He X., Yang F., Zhang Z. (2023). Interaction of cerium oxide nanoparticles and ionic cerium with duckweed (*Lemna minor* L.): Uptake, distribution and phytotoxicity. Nanomaterials.

[75] Lee H., De Saeger J., Bae S., Kim M., Depuydt S., Heynderickx P.M., Wu D., Han T., Park J. (2023). 2023. Giant duckweed (*Spirodela polyrhiza*) root growth as a simple and sensitive indicator of copper and chromium contamination. Toxics.

[76] Rozman U., Turk T., Skalar T., Zupancic M., Korosi N.C., Marinsek M., Olivero-Verbel J., Kalcikova G. (2021). An extensive characterization of various environmentally relevant microplastics—Material properties, leaching and ecotoxicity testing. Sci. the Total Environ..

[77] Olah V., Hepp A., Mesaros I. (2015). Comparative study on the sensitivity of turions and active fronds of giant duckweed (*Spirodela polyrhiza* (L.) Schleiden) to heavy metal treatments. Chemosphere.

[78] Olah V., Hepp A., Meszaros I. (2016). Assessment of Giant Duckweed (*Spirodela polyrhiza* L. Schleiden) turions as model objects in ecotoxicological applications. Bull. Environ. Contam. Toxicol..

[79] (2023). Water Quality—Aquatic Toxicity Test Based on Root Regrowth in Lemna Minor.

[80] Ziegler P. (2024). The developmental cycle of *Spirodela polyrhiza* turions: A model for turion-based duckweed overwintering?. Plants.

[81] (2017). Water Quality—Determination of the Growth Inhibition Effects of Waste Waters, Natural Waters and Chemicals on the Duckweed *Spirodela polyrhiza*—Method Using a Stock Culture Independent Microbiotest.

[82] Brain R.A., Cedergreen N. (2009). Biomarkers in aquatic plants: Selection and utility. Rev. Environ. Contam. Toxicol..

[83] Pietrini F., Passatore L., Fischetti E., Carloni S., Ferrario C., Polesello S., Zacchini M. (2019). Evaluation of morpho-physiological traits and contaminant accumulation ability in *Lemna minor* L. treated with increasing perfluorooctanoic acid (PFOA) concentrations under laboratory conditions. Sci. Total Environ..

[84] Aliferis K.A., Materzok S., Paziotou G.N., Chrysayi-Tokousbalides M. (2009). *Lemna minor* L. as a model organism for ecotoxicological studies performing ^1^H NMR fingerprinting. Chemosphere.

[85] Lomagin A.G., Ulyanova L.V. (1993). A new bioassay on water pollution using duckweed *Lemna minor* L.. Soviet Plant Physiol..

[86] Yang J., Zhao X., Wang X., Xia M., Ba S., Lim B.L., Hou H. (2024). Biomonitoring of heavy metals and their phytoremediation by duckweeds: Advances and prospects. Environ. Res..

[87] Li T., Xiong Z. (2004). A novel response of wild-type duckweed (*Lemna paucicostata* Hegelm.) to heavy metals. Environ. Toxicol..

[88] Henke R., Eberius M., Appenroth K.-J. (2011). Induction of frond abscission by metals and other toxic compounds in *Lemna minor*. Aquat. Toxicol..

[89] Olah V., Hepp A., Lakatos G., Meszaros I. (2014). Induced turion formation of *Spirodela polyrhiza* (L.) Schleiden. Acta Biol. Szeged..

[90] Appenroth K.-J., Krech K., Keresztes A., Fischer W., Koloczek H. (2010). Effects of nickel on the chloroplasts of the duckweeds *Spirodela polyrhiza* and *Lemna minor* and their possible use in biomonitoring and phytoremediation. Chemosphere.

[91] Sree K.S., Keresztes A., Mueller-Roeber B., Brandt R., Eberius M., Fischer W., Appenroth K.-J. (2015). Phytotoxicity of cobalt ions on the duckweed *Lemna minor*—Morphology, ion uptake and starch accumulation. Chemosphere.

[92] Baciak M., Sikorski L., Piotrowicz-Cieslak A.I., Adomas B. (2016). Content of biogenic amines in *Lemna minor* (common duckweed) growing in medium contaminated with tetracycline. Aquat. Toxicol..

[93] Elbasan F., Arikan-Abdulveli B., Ozfidan-Konakci C., Yildiztugay E., Tarhan I., Celik B. (2024). Exploring the defense strategies of benzalkonium chloride exposure on the antioxidant system, photosynthesis and ROS accumulation in *Lemna minor*. Chemosphere.

[94] Olah V., Hepp A., Irfan M., Meszaros I. (2021). Chlorophyll fluorescence imaging-based duckweed phenotyping to assess acute phytotoxic effects. Plants.

[95] Küster A., Altenburger R. (2007). Development and validation of a new fluorescence-based bioassay for aquatic macrophyte species. Chemosphere.

[96] Küster A., Pohl K., Altenburger R. (2007). A fluorescence-based bioassay for aquatic macrophytes and its suitability for effect analysis of non-photosystem II inhibitors. Environ. Sci. Pollut. Res..

[97] Pietrini F., Passatore L., Carloni S., Zacchini M., Aftab T. (2023). Non-standard physiological endpoints to evaluate the toxicity of emerging contaminants in aquatic plants: A case study on the exposure of *Lemna minor* and *Spirodela polyrhiza* (L.) Schleid. to dimethyl phthalate (DMP). Emerging Contaminants and Plants. Emerging Contaminants and Associated Treatment Technologies.

[98] Razinger J., Drinovec L., Zrimec A. (2007). Real-time in vivo visualization of oxidative stress in duckweed (*Lemna minor* L.). Cent. Eur. J. Biol..

[99] Persic V., Derd T., Varga M., Hackenberger B.K. (2019). Real-time CO_2_ uptake/emission measurements as a tool for early indication of toxicity in Lemna-tests. Aquat. Toxicol..

[100] Kumar S., Barth A. (2010). Following enzyme activity with infrared spectroscopy. Sensors.

[101] Schwaighofer A., Akhgar C.K., Lendl B. (2021). Broadband laser-based mid-IR spectroscopy for analysis of proteins and monitoring of enzyme activity. SAA.

[102] Pietrini F., Zacchini M. (2020). A new ecotoxicity assay for aquatic plants; Eco-Tox Photosystem Tool (ETPT). Trends Plant Sci..

[103] Wang W., Li R., Zhu Q., Tang X., Zhao Q. (2016). Transcriptomic and physiological analysis of common duckweed *Lemna minor* responses to NH_4_^+^ toxicity. BMC Plant Biol..

[104] Xu H., Yu C., Xia X., Li M., Li H., Wang Y., Wang S., Wang C., Ma Y., Zhou G. (2018). Comparative transcriptome analysis of duckweed (*Landoltia punctata*) in response to cadmium provides insights into molecular mechanisms underlying hyperaccumulation. Chemosphere.

[105] Muthan B., Wang J., Welti R., Kosma D.K., Yu L., Deo B., Khatiwada S., Vulavala V.K.R., Childs K.L., Xu C. (2024). Mechanisms of *Spirodela polyrhiza* tolerance to FGD wastewater-induced heavy-metal stress: Lipidomics, transcriptomics, and functional validation. J. Hazard. Mater..

[106] Loll A., Reinwald H., Ayobahan S.U., Göckener B., Salinas G., Schäfers C., Schlich K., Hamscher G., Eilebrecht S. (2022). Short-term test for toxicogenomic analysis of ecotoxic modes of action in *Lemna minor*. Environ. Sci. Technol..

[107] Su C., Jiang Y., Yang Y., Zhang W., Xu Q. (2019). Responses of duckweed (*Lemna minor* L.) to aluminum stress: Physiological and proteomics analyses. Ecotoxicol. Environ. Saf..

[108] Kostopoulou S., Ntatsi G., Arapis G., Aliferis K.A. (2020). Assessment of the effects of metribuzin, glyphosate, and their mixtures on the metabolism of the model plant *Lemna minor* L. applying metabolomics. Chemosphere.

[109] Wahman R., Cruzeiro C., Graßmann J., Schröder P., Letzel T. (2022). The changes in *Lemna minor* metabolomic profile: A response to diclofenac incubation. Chemosphere.

[110] Hu L.-X., Ying G.-G., Chen X.-W., Huang G.-Y., Liu Y.-S., Jiang Y.-X., Pan C.-G., Tian F., Martin F.L. (2017). Fourier transform infrared spectroscopy as a novel approach to providing effect-based endpoints in duckweed toxicity testing. Environ. Toxicol. Chem..

[111] Wu Y.-L., Xiong Q., Wang B., Liu Y.-S., Zou P.-L., Hu L.-X., Liu F., Ying G.-G. (2023). Screening of structural and functional alterations in duckweeds (*Lemna minor*) induced by per- and polyfluoroalkyl substances (PFASs) with FTIR spectroscopy. Environ. Pollut..

[112] Zhang T., Lu Q., Su C., Yang Y., Hu D., Xu Q. (2017). Mercury induced oxidative stress, DNA damage, and activation of antioxidative system and Hsp70 induction in duckweed (*Lemna minor*). Ecotoxicol Environ. Saf..

[113] Brkanac S.R., Domijan A.-M., Stefanic P.P., Maldini K., Sikiric M.D., Bol V.V., Cvjetko P. (2024). Difference in the toxic effects of micro and nano ZnO particles on *L. minor*—An integrative approach. Environ. Sci. Pollut. Res..

[114] Pietrini F., Iannilli V., Passatore L., Carloni S., Sciacca G., Cerasa M.M., Zacchini M. (2022). Ecotoxicological and genotoxic effects of dimethyl phthalate (DMP) on *Lemna minor* L. and *Spirodela polyrhiza* (L.) Schleid. plants under a short-term laboratory assay. Sci. Total Environ..

[115] Ma X., Jiang Y., Qu Z., Yang Y., Wang W., He Y., Yu Y., Luo X., Liu W., Han W. (2024). Overexpression of phosphoserine aminotransferase (*PSAT*)-enhanced cadmium resistance and accumulation in duckweed (*Lemna turionifera* 5511). Plants.

[116] Van Hoeck A., Horemans N., Nauts R., VanHees M., Vandenhove H., Blust R. (2017). *Lemna minor* plants chronically exposed to ionizing radiation: RNA-seq analysis indicates a close rate dependent shift from acclimation to survival strategies. Plant Sci..

[117] Fu L., Ding Z., Sun X., Zhang J. (2019). Physiological and transcriptomic analysis reveals distorted ion homeostasis and response in the freshwater plant *Spirodela polyrhiza* L. under salt stress. Genes.

[118] Shang S., Zhang Z., Li L., Chen J., Zang Y., Liu X., Wang J., Tang X. (2023). Transcriptome analysis reveals gene expression patterns of *Spirodela polyrhiza* response to heat stress. Int. J. Biol. Macromol..

[119] Yu C., Zhao X., Qi G., Bai Z., Wang Y., Wang S., Ma Y., Liu Q., Hu R., Zhou G. (2017). Integrated analysis of transcriptome and metabolites reveals an essential role of metabolic flux in starch accumulation under nitrogen starvation in duckweed. Biotechnol. Biofuels.

[120] Fu L., Tan D., Sun X., Ding Z., Zhang J. (2020). Transcriptional analysis reveals potential genes and regulatory networks involved in salicylic acid-induced flowering in duckweed *(Lemna gibba*). Plant. Physiol. Biochem..

[121] Wang W., Wu Y., Messing J. (2014). RNA-Seq transcriptome analysis of Spirodela dormancy without reproduction. BMC Genom..

[122] Pasaribu B., Acosta K., Aylward A., Liang Y., Abramson B.W., Colt K., Hartwick N.T., Shanklin J., Michael T.P., Lam E. (2023). Genomics of turions from the Greater Duckweed reveal its pathways for dormancy and re-emergence strategy. New Phytol..

[123] Wang W., Yang C., Tang X., Gu X., Zhu Q., Pan K., Hu Q., Ma D. (2014). Effects of high ammonium level on biomass accumulation of common duckweed *Lemna minor* L.. Environ. Sci. Pollut. Res..

[124] Afshana, Dyar M.A., Reshi Z.A., Khan Z., Ansari M.Y.K., Shahwar D. (2021). Induced genotoxicity and oxidative stress in plants: An overview. Induced Genotoxicity and Oxidative Stress in Plants.

[125] Picinini-Zambelli J., Garcia A.L.H., Da Silva J. (2025). Emerging pollutants in the aquatic environment: A review of genotoxic impacts. MRR.

[126] Kaur L., Kanwar N. (2022). Duckweed: A model for phytoremediation technology. Holist. Approach Environ..

[127] Ekperusi A.O., Sikoki F.D., Nwachukwu E.O. (2019). Application of common duckweed (*Lemna minor*) in phytoremediation of chemicals in the environment; State and future perspective. Chemosphere.

[128] Ekperusi A.O., Sikoki F.D., Nwachukwu E.O. (2023). Sorption of cadmium, chromium, lead, and vanadium from artificial wetlands using *Lemna aequinoctialis*. Int. J. Phytoremed..

[129] Yang L., Yao J., Sun J., Shi L., Cen Y., Su J. (2020). The Ca^2+^ signaling, Glu, and GABA responds to Cd stress in duckweed. Aquat. Toxicol..

[130] Yang L., Han H., Liu M., Zuo Z., Zhou K., Lü J., Zhu Y., Bai Y., Wang Y. (2013). Overexpression of the *Arabidopsis* photorespiratory pathway gene, serine: Glyoxylate aminotransferase (*AtAGT1*), leads to salt stress tolerance in transgenic duckweed (*Lemna minor*). Plant Cell. Tiss. Organ Cult..

[131] Canto-Pastor A., Molla-Morales A., Ernst E., Dahl W., Zhai J., Yan Y., Meyers B.C., Shanklin J., Martienssen R. (2015). Efficient transformation and artificial miRNA gene silencing in *Lemna minor*. Plant Biol..

[132] Gan W.C., Ling A.P.K. (2022). CRISPR/Cas9 in plant biotechnology: Applications and challenges. BioTechnologia.

[133] Liu Y., Wang Y., Xu S., Tang X., Zhao J., Yu C., He G., Xu H., Wang S., Tang Y. (2019). Efficient genetic transformation and CRISPR/Cas9-mediated genomic editing in *Lemna aequinoctialis*. Plant Biotechnol. J..

